# Quantitative analyses of products and rates in polyethylene depolymerization and upcycling

**DOI:** 10.1016/j.xpro.2023.102575

**Published:** 2023-09-19

**Authors:** Yu-Hsuan Lee, Jiakai Sun, Susannah L. Scott, Mahdi M. Abu-Omar

**Affiliations:** 1 Department of Chemistry and Biochemistry, University of California, Santa Barbara, Santa Barbara, CA 93106, USA; 2 Department of Chemical Engineering, University of California, Santa Barbara, Santa Barbara, CA 93106, USA

**Keywords:** NMR, Chemistry, Material Sciences, Environmental Sciences

## Abstract

Depolymerization and upcycling are promising approaches to managing plastic waste. However, quantitative measurements of reaction rates and analyses of complex product mixtures arising from depolymerization of polyolefins constitute significant challenges in this emerging field. Here, we detail techniques for recovery and analysis of products arising from batch depolymerization of polyethylene. We also describe quantitative analyses of reaction rates and products selectivity. This protocol can be extended to depolymerization of other plastics and characterization of other product mixtures including long-chain olefins.

For complete details on the use and execution of this protocol, please refer to Sun et al.^1^

## Highlights

• Techniques for product recovery of polyethylene depolymerization
• Characterization and quantification of hydrocarbon products in complex mixtures
• Quantitative analysis of reaction selectivity for alkanes versus aromatics
• Enumeration of depolymerization rates using average rates of C–C bond scission

## Abstract (editor highlights)

Publisher’s note: Undertaking any experimental protocol requires adherence to local institutional guidelines for laboratory safety and ethics.

## Graphical abstract

**Figure:**
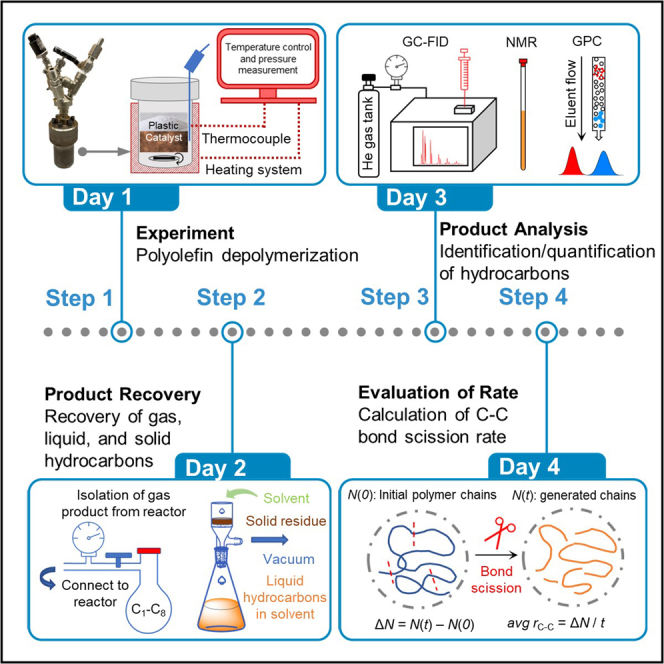

## Before you begin

Two polyolefins, polyethylene (PE) and polypropylene (PP), comprise half of the annual global plastic production.^2^ Due to their extensive use in single-use products, they also account for half of the resulting plastic waste.^2^ Depolymerization of polyolefins is challenging because of the thermodynamic strength and low reactivity of the non-polar C-C bonds, as well as the absence of chemical functionality in the polymers. Pyrolysis, practiced at elevated temperatures (typically ≥ 400°C), results in a complex pyrolytic oil, as well as a large amount of non-volatiles and insoluble char.^3^ Molecular recycling/upcycling of polyolefins at milder temperatures can yield more useful and more targeted distributions of hydrocarbon products. Catalytic hydrogenolysis and/or hydrocracking of polyolefins in the presence of excess H_2_ produces liquid hydrocarbons that may be suitable for use as fuels and lubricants.^4^^,^^5^ Tandem hydrogenolysis/aromatization at ≤ 300°C without added H_2_ can generate valuable long-chain alkylaromatics,^6^ which may be used to manufacture anionic surfactants.^7^ Although the process generated a narrow molecular weight distribution (carbon number average C_∼30_, *Ð* = 1.1) relative to the starting polyethylene (carbon number average C_∼132_, *Ð* = 1.9),^6^ the products were a complex mixture of hydrocarbons which may include *n*-alkanes, *iso*-alkanes, naphthenes, alkyltetralins, alkylbenzenes, alkylnaphthalenes, alkylphenanthrenes, and other polycyclic aromatics.

The challenges involved in analyzing such mixtures are both qualitative and quantitative. To characterize hydrocarbons ranging from C_1_ to C_∼40_, gas chromatography (GC) is typically used to separate components based primarily on their boiling points, followed by mass spectrometry (MS) or flame ionization detection (FID).^8^^,^^9^ Due to the diversity in hydrocarbon product structures associated with similar physical properties, 1D separation may not be sufficient. When available, 2D GC x GC with polarity separation as an additional dimension can be used to improve the separation.^10^^,^^11^ Since hydrocarbons are susceptible to extensive fragmentation under electron ionization (EI), information about the mass of the parent ion is often lost with conventional GC-EIMS. Alternatives include soft ionization techniques including matrix-assisted laser desorption ionization (MALDI),^12^ field desorption (FD),^6^ and nitric oxide ionization spectrometry evaluation (NOISE)^13^ where the masses of the molecular ions are desired. For mixtures that include heavier hydrocarbons (C_>40_), gel permeation chromatography (GPC) can be used to assess the molecular weight distribution.^14^ However, the limited solubility of the hydrocarbons in GPC solvents may necessitate special equipment in the form of high temperature GPC. Alternatively, insoluble hydrocarbons can be characterized using MALDI-MS or FD-MS. In many cases, analysis is barely attempted, and catalytic depolymerization activity is simply reported as the mass yields of gas, liquid and solid hydrocarbons. Since these categories are poorly defined, they can be misleading when used to compare and rank catalysts and reaction conditions.

Here, we describe a comprehensive approach to obtaining quantitative rates for partial polyethylene depolymerization. It involves inferring the number of C-C bond scission events from the molecular weight distribution, in combination with product yields. Methods for estimating yields and selectivities are presented. Specifically, we quantify the alkylaromatics in complex mixtures of hydrocarbons (alkanes and alkylaromatics) derived from polyethylene. While the reaction kinetics are complex and apparent rate constants do not represent elementary reaction steps, measuring average C-C bond scission rates can facilitate direct comparison of catalysts and the effect of reaction conditions, accelerating catalyst design and discovery.

## Key resources table

**Table:** 

REAGENT or RESOURCE	SOURCE	IDENTIFIER
**Chemicals**		

Polyethylene (*M*_w_ = 3.5 × 10^3^ g mol^–1^, *Ð* = 1.9, ∼46 methyl groups/1000 carbons)	Sigma-Aldrich	CAS#9002-88-4
Pt/F-Al_2_O_3_ (1.6 wt % Pt, 0.8 wt % F)	Synthesized according to Sun et al.^1^	
Pt/Cl-Al_2_O_3_ (1.5 wt % Pt, 1.4 wt % Cl)	Synthesized according to Sun et al.^1^	
Propene (99.8%)	Praxair	CAS#115-07-1
Dichloromethane (≥ 99.5%)	Fisher Scientific	CAS#75-09-2
Methane (UHP)	Airgas	CAS#74-82-8
Ethane (≥ 99.95%)	Sigma-Aldrich	CAS#74-84-0
Propane (99.99%)	Praxair	CAS#74-98-6
*norm*-butane (99%)	Sigma-Aldrich	CAS#106-97-8
*norm*-pentane (≥99%)	Sigma-Aldrich	CAS#109-66-0
*norm*-hexane (95%)	Sigma-Aldrich	CAS#110-54-3
*norm*-heptane (99%)	Sigma-Aldrich	CAS#142-82-5
*norm*-octane (≥ 99%)	Sigma-Aldrich	CAS#111-65-9
Standard mixture of saturated alkanes (C_7_-C_40_, certified reference material, 1000 μg/mL each component in hexane)	Sigma-Aldrich	SKU: 49452-U
Chromium(III) acetylacetonate (99.99%)	Sigma-Aldrich	CAS#21679-31-2
HPLC-grade hexanes	Fisher Scientific	CAS#110-54-3
Triethylamine (≥ 99.5%)	Fisher Scientific	CAS#121-44-8
Chloroform-*d* (D, 99.8%)	Cambridge Isotope Laboratories	CAS#865-49-6
1,1,2,2-tetrachloroethane-*d*_2_ (D, 99.5%)	Cambridge Isotope Laboratories	CAS#33685-54-0
Argon (UHP)	Airgas	CAS#7440-37-1

**Software and algorithms**		

Mestrenova	Mestrelab Research	https://mestrelab.com/
Origin 2022	OriginLab	https://www.originlab.com/

**Other**		

Stir bar, Pyrex (22 × 6.4 mm)	VWR	https://us.vwr.com/
Dewar flask (500 mL)	Pope Scientific Inc.	https://www.popeinc.com/
Sieve (250 μm, 425 μm)	Endecotts	https://www.endecotts.com/
Syringe filter (PTFE, 13 mm, 0.2 μm)	Agilent	https://www.agilent.com/
Plastic syringe (1 mL)	Covidien	https://www.medtronic.com/covidien/
Luer lock gas-tight syringe	VICI Precision Sampling	https://www.vici.com/index.php/
Glovebox (argon atmosphere)	MBRAUN	https://www.mbraun.com/
Rotary vane pump	Edwards RV3	https://www.edwardsvacuum.com/
Stainless steel autoclave	Parr Series 5000 Multiple Reactor System	https://www.parrinst.com/
Gas chromatograph (GC)	Shimadzu GC-2010 gas chromatograph	https://www.shimadzu.com/
Gas chromatograph (GC)	Agilent 6890N network gas chromatograph	https://www.agilent.com/
Rotary evaporator	Rotavapor R-100, heating bath B-100, interface I-100, vacuum pump V-100	https://www.buchi.com/
Gel permeation chromatograph (GPC)	Agilent PL-GPC 220 gel permeation chromatograph	https://www.agilent.com/
Gel permeation chromatograph (GPC)	Waters Alliance HPLC System (2690 Separation Module)	https://www.waters.com/ SKU: 21327
Nuclear magnetic resonance spectrometer (solution-state NMR)	Bruker Avance NEO	https://www.bruker.com/ 500 MHz
Nuclear magnetic resonance spectrometer (solution-state NMR)	Varian Unity Inova AS600	600 MHz
UV-visible spectrometer (UV-vis)	Shimadzu UV-2401PC spectrophotometer	https://www.shimadzu.com/
Thermogravimetric analyzer (TGA)	TA Discovery thermogravimetric analyzer	https://www.tainstruments.com/

***Note:*** All chemicals were used as received without further purification.

***Note:*** Store air-sensitive catalysts such as Pt/F-Al_2_O_3_ and Pt/Cl-Al_2_O_3_ in an inert atmosphere glovebox.

**CRITICAL:** Methylene chloride, chloroform-*d*, and 1,1,2,2-tetrachloroethane-*d*_2_ are acutely toxic and are volatile organic compounds. Keep the containers tightly closed, store in a flammable storage cabinet, and use only in a fume hood.

## Materials and equipment

**Table:** 

Instruments/Techniques	Equipment
Stainless steel autoclave	90 mL in volume, equipped with a 250-watt external heater
Gas Chromatography (for gas product analysis, C_1_-C_8_)	GC, equipped with a flame ionization detector (FID), Agilent DB-1 column
Gas Chromatography (for volatile liquid product analysis, C_7_-C_11_)	GC, equipped with an FID detector, Agilent DB-5 column
Gel Permeation Chromatography	GPC, equipped with a refractive index (RI) detector, calibrated with Varian monomodal, linear PE standards. Columns: PL-Gel Mixed B Guard column, three PL-Gel Mixed B columns.
Gel Permeation Chromatography	GPC, equipped with Waters 2410 RI detector and Waters 2998 photodiode array detector (PDA), calibrated with polystyrene standards (Agilent EasiVial kit, molecular weights in the range of 200–400,000 g mol^−1^). Columns: two PL-Gel MiniMIX-D column, a guard column.

For detailed information regarding instrument parameters, please refer to Sun et al.^1^

## Step-by-step method details

In this section, we describe in detail how to accomplish polyethylene depolymerization and the steps for recovery and quantification of hydrocarbon reaction products.

### Polyethylene depolymerization

**Timing: Variable – 24 h for our study**

Depolymerization was carried out in a batch reactor at a set temperature under an inert atmosphere.

1. Prior to each reaction, place a stainless-steel autoclave (90 mL), its lid and a Pyrex-encapsulated stir bar into the antechamber of an Ar-filled glovebox and evacuate for 12 h, then bring the autoclave into the glovebox.
2. Inside the glovebox, load the stir bar, the desired masses of polyethylene (typically, 0.120 g), and a freshly reduced Pt catalyst (0.200 g, Pt/F-Al_2_O_3_ as an example) into the autoclave.
  a. Mix the solids with a spatula.
  b. Seal and remove the reactor from the glovebox.

***Note:*** The solid catalyst can be pre-activated under H_2_ (please refer to Sun et al.)^1^ and stored in the glovebox prior to use.

***Note:*** To accelerate the initial mass transport of PE and promote heat transfer to the polymer, grind and sieve the polymer into a fine powder (250–425 μm) before loading it into the reactor.

Cryogenic grinding: Under air, place the polymer beads (5–10 g) into a clean Dewar flask (500 mL). Next, add enough liquid N_2_ to cover the beads. Allow the majority of the liquid N_2_ to boil off, then promptly pour the beads into a coffee grinder and grind for a few seconds to obtain polymer powder.

**Figure 1:**
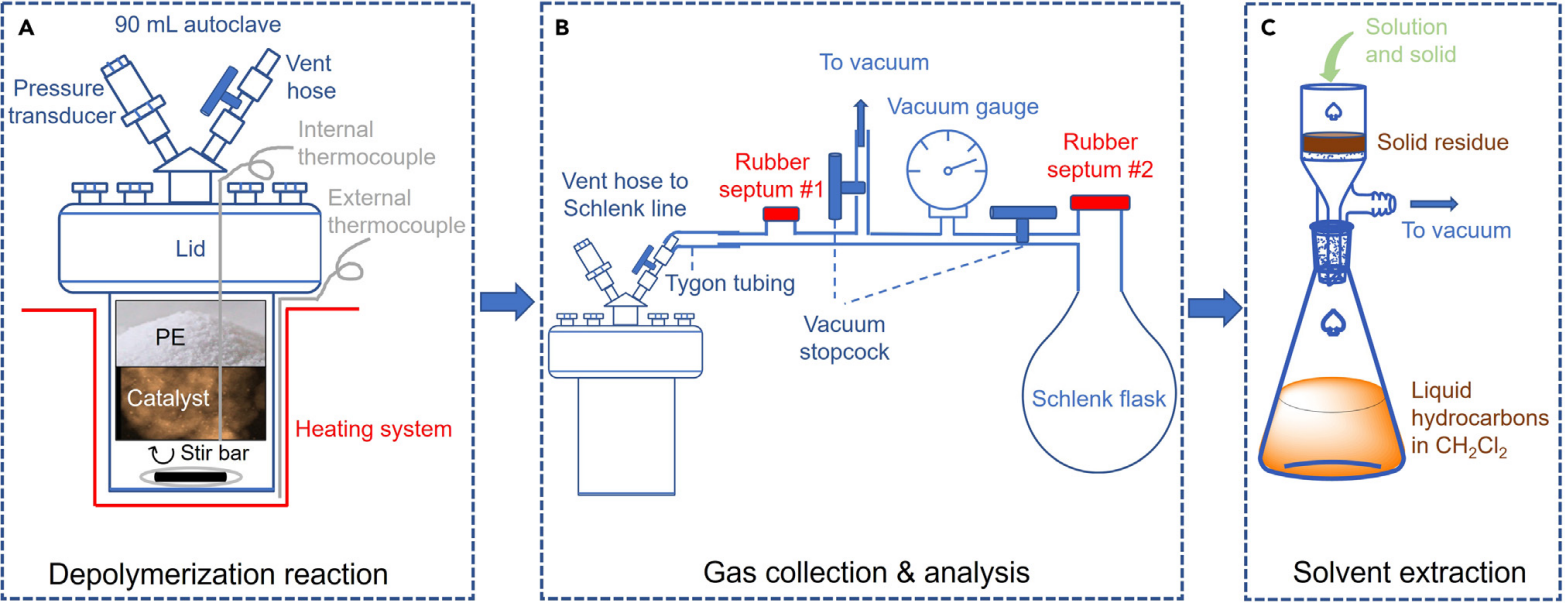
Schematic representation of depolymerization reaction and product recovery Schematic representation of (A) depolymerization batch reaction, (B) post-reaction gas collection, and (C) post-reaction solvent extraction of soluble hydrocarbons and solid recovery.
  b. Start the reaction timer when the reactor reaches the set temperature (∼25 min).

***Note:*** If the internal thermocouple does not touch the polymer/catalyst mixture, it may measure the internal gas temperature rather than the temperature of the solid/liquid phase. We also used a thermocouple to measure the external temperature of the autoclave wall and found that, typically, the temperature of the reactor wall is 280 ± 5°C, i.e., 30°C higher than that of the gas inside the reactor).

***Note:*** The temperature at which the depolymerization reaction occurs relies on the measurement of the external temperature of the reactor. For autoclaves that do not contain an internal thermocouple, the desired temperature can be set through the external heating system and the temperature can be measured externally.

3. Place the sealed reactor inside an external vessel heater, insert a thermocouple through a feedthrough into the reactor.
  a. Set the desired temperature, stirring rate, and reaction time (typically, 250°C, 675 rpm, 8 h, Figure 1A).

4. After the desired reaction time, remove the reactor from the vessel heater and quench the reaction by immersing the reactor vessel in a water bath at 20°C–25°C for ca. 30 min.

### Recovery and quantification of hydrocarbon reaction products

**Timing: ∼12 h for workup and analyses**

These steps describe the recovery and quantification of each class of hydrocarbon products (gas, liquid, and solid) generated by polyethylene depolymerization.

5. Connect the autoclave and a Schlenk flask (typically, 100 mL) capped with rubber septum #2 to a Schlenk line equipped with a vacuum gauge and rubber septum #1, as shown in Figure 1B.
6. Evacuate the Schlenk flask and line (≤ 10^−2^ Torr), then isolate both from the vacuum pump.
7. Expand the gases from the autoclave headspace into the line and Schlenk flask. Isolate the Schlenk flask.

***Note:*** Wait an additional minute or so after the pressure reading stabilizes in the Schlenk line before closing the Schlenk flask, to ensure all gas components are well-mixed.

**CRITICAL:** Ensure the pressure of the line and the Schlenk flask will be lower than 1 atm after gas expansion, to avoid ejecting the rubber septum. The pressure in the reactor, post-reaction, can be measured using a pressure transducer, and this value can be used with the ideal gas law to estimate the pressure that will be generated in the line and the Schlenk flask after gas expansion. Adjust the size of the Schlenk flask so that the pressure after expansion does not exceed 1 atm.

8. Remove an aliquot of gas (400 μL) via rubber septum #1 (Figure 1B) using a Luer lock gas-tight syringe,
  a. Inject the aliquot into the GC-FID to record the initial chromatogram.
  b. Perform this step twice to ensure consistency of the result.
9. Inject propene (400 μL, 200–400 mbar) as internal standard into the Schlenk flask via rubber septum #2 (Figure 1B).

***Note:*** Pull and push the syringe plunger several times to ensure good mixing between the internal standard and the gases in the Schlenk flask.

10. Remove an aliquot (200 μL) of gas from the Schlenk flask using a Luer lock gas-tight syringe.
  a. Inject into the GC-FID for light hydrocarbon analysis (C_1_-C_8_).
    ***Note:*** The difference in areas for the propene peaks in both chromatograms can be used to calculate the amount of propene present as a reaction product, relative to the amount added as an internal standard.
  b. Perform this step twice to ensure well-mixing of the gaseous products and the internal standard.
11. Disconnect the reactor vent hose from the Schlenk line. Prior to opening the reactor, add 5 mL of methylene chloride (in 5× 1 mL aliquots) via the vent hose (Figure 1B).

***Note:*** This step ensures that any liquid hydrocarbons that have condensed in the upper part of the reactor including the reactor’s lid will dissolve and combine with the liquids present at the bottom of the reactor vessel.

12. Open the reactor, allow the CH_2_Cl_2_ to enter, and transfer the solution and solid using a glass Pasteur pipette onto a Buchner filter funnel equipped with a fine glass frit (4.0–5.5 μm).
13. Add another 5 mL CH_2_Cl_2_ to wash the solid remaining on the frit to increase the recovery of liquid hydrocarbons adsorbed on the solid residue in the filtrate.
14. Transfer the filtrate to a volumetric flask and dilute to 10.00 mL volume using CH_2_Cl_2_.
  a. Filter ∼1 mL of the solution through a 0.2 μm PTFE filter attached to a 1 mL plastic syringe for analysis by GC-FID.
15. Evaporate the majority of the CH_2_Cl_2_ solvent using rotary evaporation (30°C, 350 Torr, 15 min), then remove residual solvent under reduced pressure on a vacuum line (0.1 Torr, 1 h) at 20°C–25°C.

***Note:*** These steps also remove volatile liquid hydrocarbons (mostly C_7_-C_11_).

16. Weigh the mass of the remaining liquid products (C_>11_).
17. Re-dissolve the liquid products in CH_2_Cl_2_ in a volumetric flask and dilute with CH_2_Cl_2_ to 10.00 mL.
  a. Filter ∼1 mL of this solution through a 0.2 μm PTFE syringe filter for analysis by GC-FID.

***Note:*** Comparison of the results from this step with those from step 14 identifies and quantifies hydrocarbons lost during solvent evaporation/removal.

18. Prepare sample analysis for ^1^H NMR, quantitative ^13^C NMR, GPC, and UV-vis (optional).
  a. Dissolve 2 mg of the liquid product in 1,1,2,2-tetrachloroethane-*d*_2_ for ^1^H NMR analysis.
  b. Dissolve 60 mg of the liquid product and 22.4 mg Cr(acac)_3_ (80 mM) in CDCl_3_ for quantitative ^13^C NMR analysis.
  c. Dissolve 6 mg of the liquid product in 1.5 mL CHCl_3_ containing 0.25 vol % triethylamine for GPC analysis.
  d. Dissolve 0.5 mg of the liquid product in HPLC-grade hexanes and dilute with hexanes to 3.00 mL in a volumetric flask. Transfer this solution into a 1-cm pathlength quartz cuvette for UV-vis measurement.
19. Add ∼10 mL CH_2_Cl_2_ to the solid mixture on the frit to create a suspension and transfer this suspension by a glass Pasteur pipette to a vial.
  a. Evaporate the majority of the CH_2_Cl_2_ solvent using rotary evaporation (30°C, 350 Torr, 15 min),
  b. Remove residual solvent under reduced pressure on a vacuum line (0.1 Torr, 1 h) at 20°C–25°C .
20. Weigh the mass of the recovered solid.
21. Perform TGA of the solid residue (∼3 mg) in air.

## Expected outcomes

In this section, we describe in detail the characterization of the hydrocarbon products formed in the catalytic conversion of PE (0.120 g, *M*_w_ = 3.5 × 10^3^ g mol^–1^, *Ð* = 1.9) over Pt/F-Al_2_O_3_ (0.200 g, 1.6 wt % Pt, 0.8 wt % F) at 280°C under Ar, as a representative example. Results for other catalysts were presented in Sun et al.^1^

After 8 h, the yields of hydrocarbon gases (C_1_-C_8_), volatile liquids (C_7_-C_11_), and heavy liquids (C_>11_) are 7 (1) , 7 (1), and 64 (1) wt %, respectively. The solid residue (12 (1) wt %) is mostly coke, based on the temperature of its oxidation according to TGA.^1^ The uncertainties are presented in parenthesis based on duplicates. Further characterization of the gas and liquid products is detailed below.

### Gas chromatographic analysis of gaseous hydrocarbons

**Figure 2:**
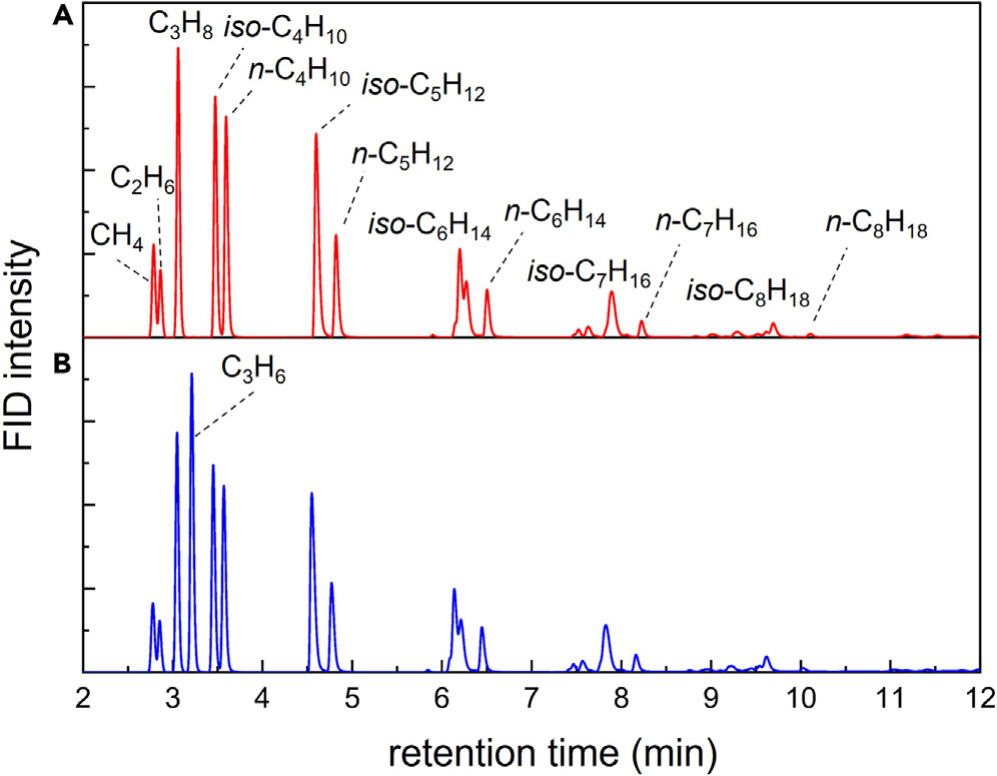
GC-FID chromatograms of gas products GC-FID chromatograms of gas products (A) before, and (B) after adding propene as an internal standard. *n*-alkanes and *iso*-alkanes were assigned based on their retention times.

GC-FID chromatograms of the gas products can be recorded before and after addition of an internal standard, shown in Figure 2. The initial chromatogram (Figure 2A) shows the presence of alkanes (C_1_-C_8_) but no propene (absence of a signal at 3.2 min), suggesting that propene is a good choice for internal standard.

### Gas chromatographic analysis of volatile liquid hydrocarbons (C_7_-C_11_)

**Figure 3:**
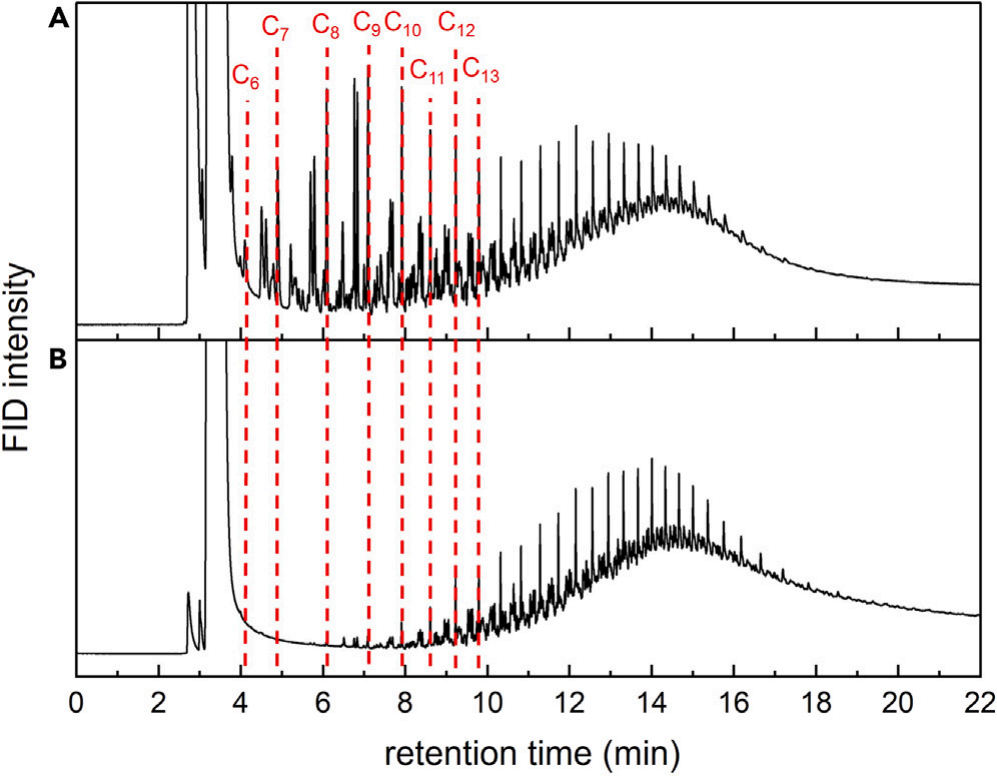
GC-FID chromatograms of liquid products GC-FID of liquid products (A) before, and (B) after removal of CH_2_Cl_2_ solvent by evaporation. The dashed lines indicate the signals for *norm*-alkanes.

GC-FID analysis of the liquid products dissolved in CH_2_Cl_2_ can be conducted with and without solvent removal. The broad, intense solvent peak obscures other signals that may be present with retention times between 2 to 4 min (Figure 3A). Comparison to the chromatogram after solvent evaporation (Figure 3B) shows that most of the hydrocarbons lost during solvent removal are in the range C_7_-C_11_, which we refer to as volatile liquid hydrocarbons.

### Quantitative NMR analysis of heavy liquid hydrocarbons (C_>11_)

**Figure 4:**
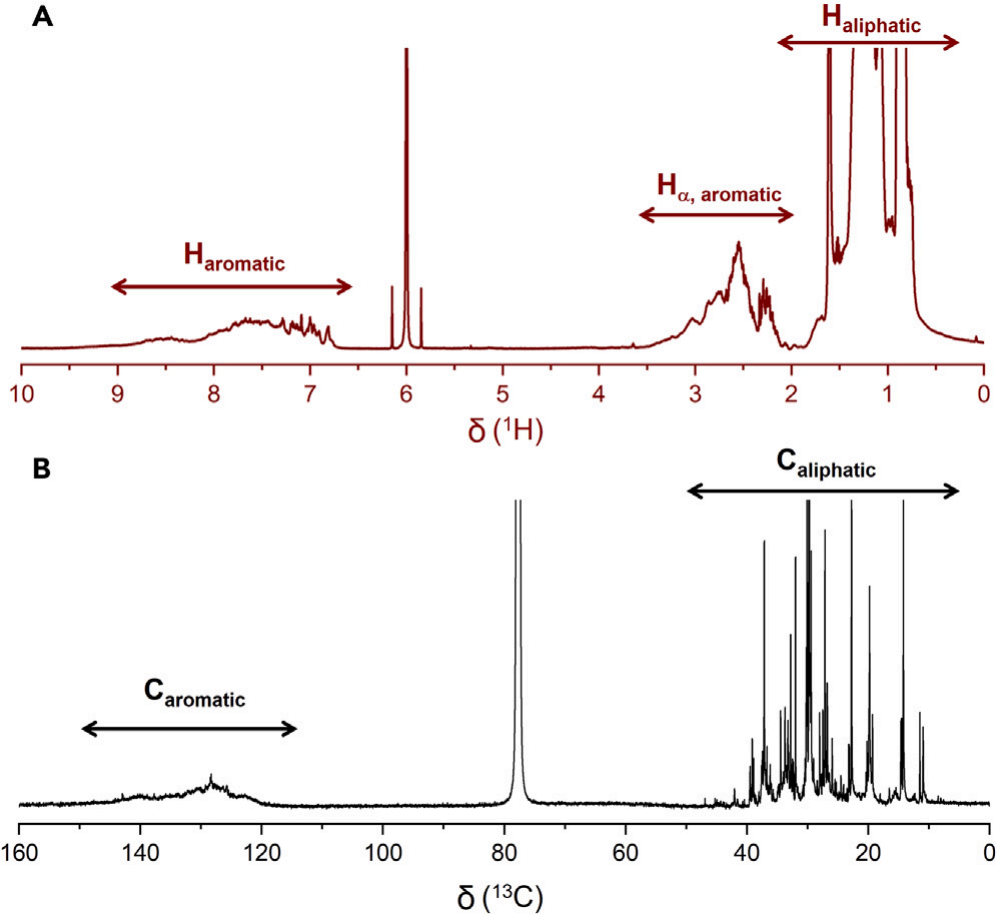
Quantitative solution-state NMR spectra of heavy liquid products (A and B) Quantitative solution-state NMR spectra of the heavy hydrocarbon liquid products (C_>11_): (A) ^1^H NMR, and (B) ^13^C NMR.

**Figure 5:**
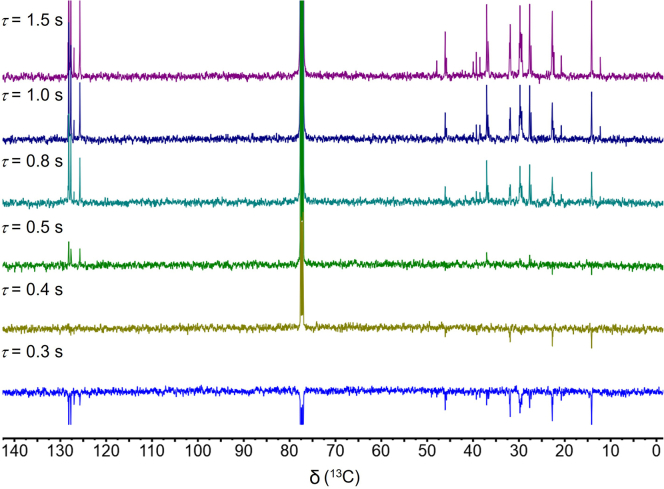
^13^C NMR spectra of dodecylbenzene, acquired using an inversion recovery pulse sequence^17^ with variable delays (*τ*) between the 180° and 90° pulses, to assess the acquisition parameters needed for quantitative measurements

Solution-state ^1^H and ^13^C NMR spectra of the liquid products can be recorded at 20°C–25°C (we used a Varian Unity Inova AS600 spectrometer and a Bruker Avance NEO 500 spectrometer for ^1^H and ^13^C NMR, respectively). A typical ^1^H NMR experiment was performed at an acquisition time of 2.5 s with 64 accumulated scans. The ^1^H NMR spectrum reveals signals for aromatic protons at 6.5–9.0 ppm and for benzylic protons (H_α_, including the benzylic positions of alkyl substituents on fused aromatics such as naphthalenes and phenanthrenes) at 2.0–3.5 ppm, indicating the presence of alkylaromatics (Figure 4A).^15^^,^^16^ To ensure that the ^13^C NMR information is quantitative, Cr(acac)_3_ is added as a relaxation agent to reduce long ^13^C spin-lattice relaxation times (*T*_*1*_). The spectrum of a model compound, dodecylbenzene (60 mg), is measured with an inversion recovery pulse sequence^17^ to choose a suitable relaxation delay (*t*) (Figure 5). An inversion recovery measurement conducted at 25°C with 80 mM Cr(acac)_3_ (22.4 mg) in CDCl_3_ (800 μL) gave approximate values for *T*_*1*_ and *t* according to Equations 1 and 2, respectively, where *τ* is the variable recovery delay between the 180° and 90° pulses.^17^

(Equation 1) $$T_{1}=\tau \;/\;\ln\;2$$

(Equation 2) $$t\geq 5\times T_{1}$$

Since all peaks in Figure 5 are positive for *τ* ≥ 0.8 s, the estimated value of *T*_*1*_ is 1.2 s. Quantitative ^13^C NMR spectra therefore require a relaxation delay *t* ≥ 6.0 s. The NMR sample of heavy liquid hydrocarbons resulting from PE depolymerization was prepared at the same Cr(acac)_3_ concentration and the *T*_1_ and *t* values determined in the inversion recovery experiment with dodecylbenzene are then used to record quantitative ^13^C NMR spectrum at an acquisition time of 1.5 s with 4096 accumulated scans. Figure 4B shows signals for aromatic carbons in the region from 118 to 150 ppm.^18^ Their integration will be used to estimate aromatic yields and selectivities (see below).

### GC-MS analysis of heavy liquid hydrocarbons (C_>11_)

**Figure 6:**
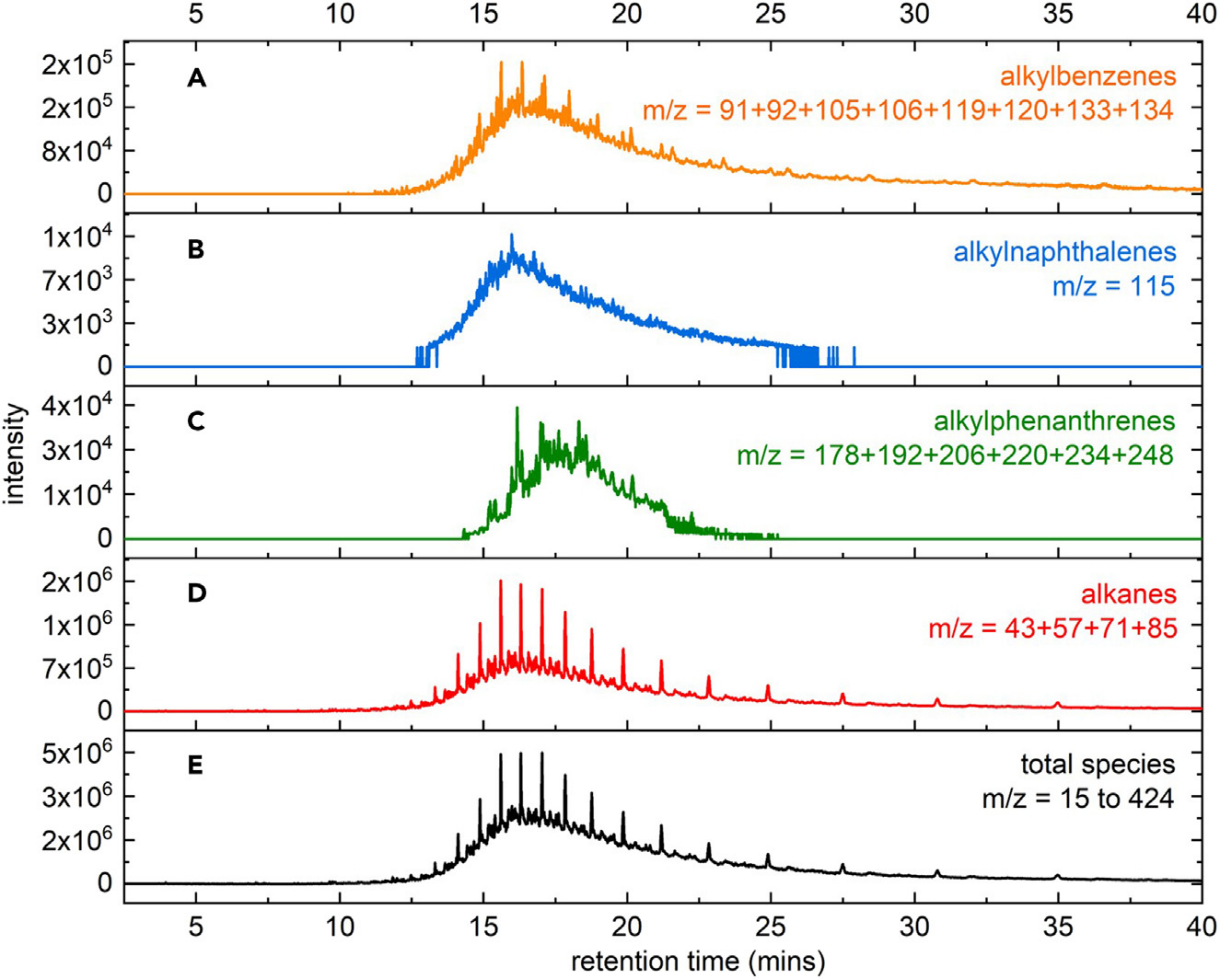
Total and characteristic ion chromatograms from GC-MS of heavy liquid products Characteristic ion chromatograms, obtained by GC-MS, for (A) alkylbenzenes, (B) alkylnaphthalenes, (C) alkylphenanthrenes, and (D) alkanes (including naphthenes), extracted from the total chromatogram (E) of a mixture of heavy liquid hydrocarbons (Figure reprinted from ref.^1^ Sun et al. (2023) with permission from Elsevier).

GC-MS can be used to determine the average carbon number for different classes of hydrocarbons present in complex mixtures. Characteristic ion chromatograms for each type of hydrocarbon (alkylbenzenes, alkylnaphthalenes, alkylphenanthrenes, and alkanes) are extracted from the total ion chromatogram, as shown in Figure 6.

### Size exclusion chromatographic analysis of heavy liquid hydrocarbons (C_>11_)

**Figure 7:**
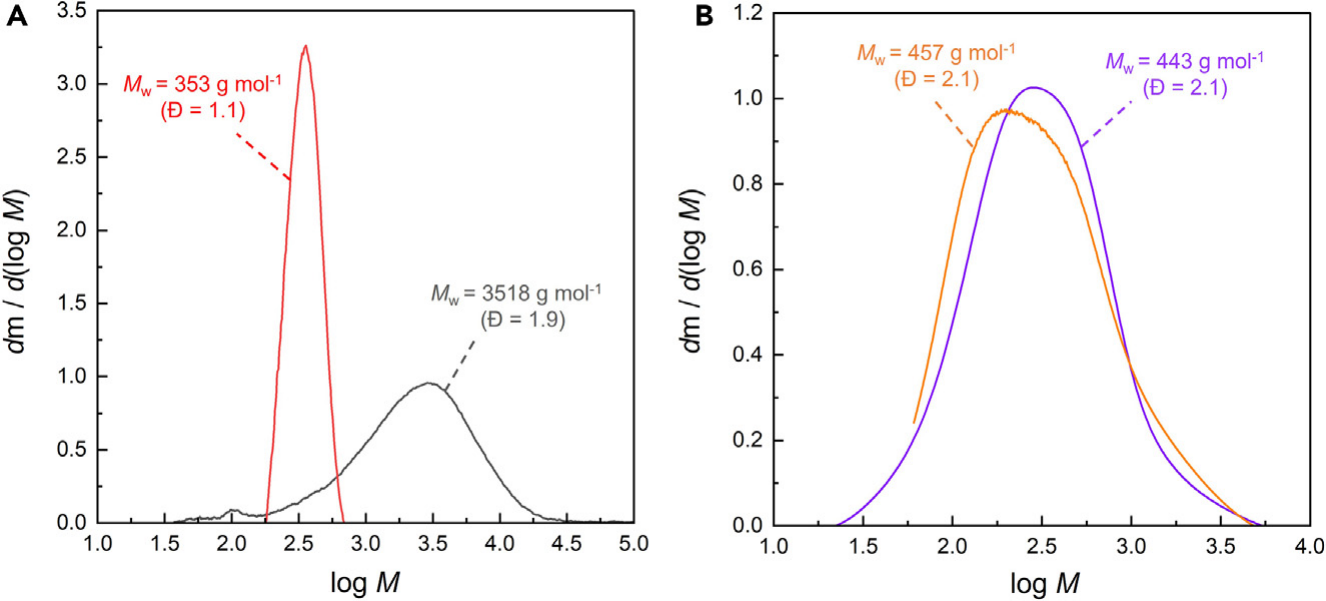
Size exclusion chromatograms of heavy liquid products (A and B) GPC analysis of (A) a low molecular weight PE (gray) and its heavy liquid depolymerization products (red), obtained with RI detection and PE standards, and (B) heavy liquid depolymerization product analyzed using both RI (purple) and UV (orange) detectors, calibrated using PS standards (Figure 7B reprinted from ref.^1^ Sun et al. (2023) with permission from Elsevier).

While PE analysis generally requires access to high temperature gel permeation chromatography (GPC), it is possible to analyze low molecular weight PE and depolymerization products using more widely available room temperature GPC, performed in a solvent such as chloroform or tetrahydrofuran. The instrument is typically equipped with RI (refractive index) and/or UV detectors. The system is usually calibrated with either PE or PS standards (the use of more readily available PS standards requires a conversion process to obtain PE molecular weights).^19^ Typical results for the heavy liquid products resulting from the conversion of PE (0.120 g, *M*_w_ = 3.5 × 10^3^ g mol^–1^, *Ð* = 1.9) catalyzed by Pt/F-Al_2_O_3_ (0.200 g, 1.6 wt % Pt, 0.8 wt % F) after 8 h at 280°C under Ar are shown in Figure 7A for a low molecular weight PE and its depolymerization products , revealing a decrease in *M*_w_ and dispersity (*Đ* = *M*_w_/*M*_n_). A comparison of results using RI and UV detectors shows how the UV-active chromophores are distributed relative to the total hydrocarbons (Figure 7B). In this case, both detection methods give similar distributions, suggesting that aromatic products are evenly distributed over the entire molecular weight range of liquid hydrocarbon products.

### Thermogravimetric analysis of solid hydrocarbons

**Figure 8:**
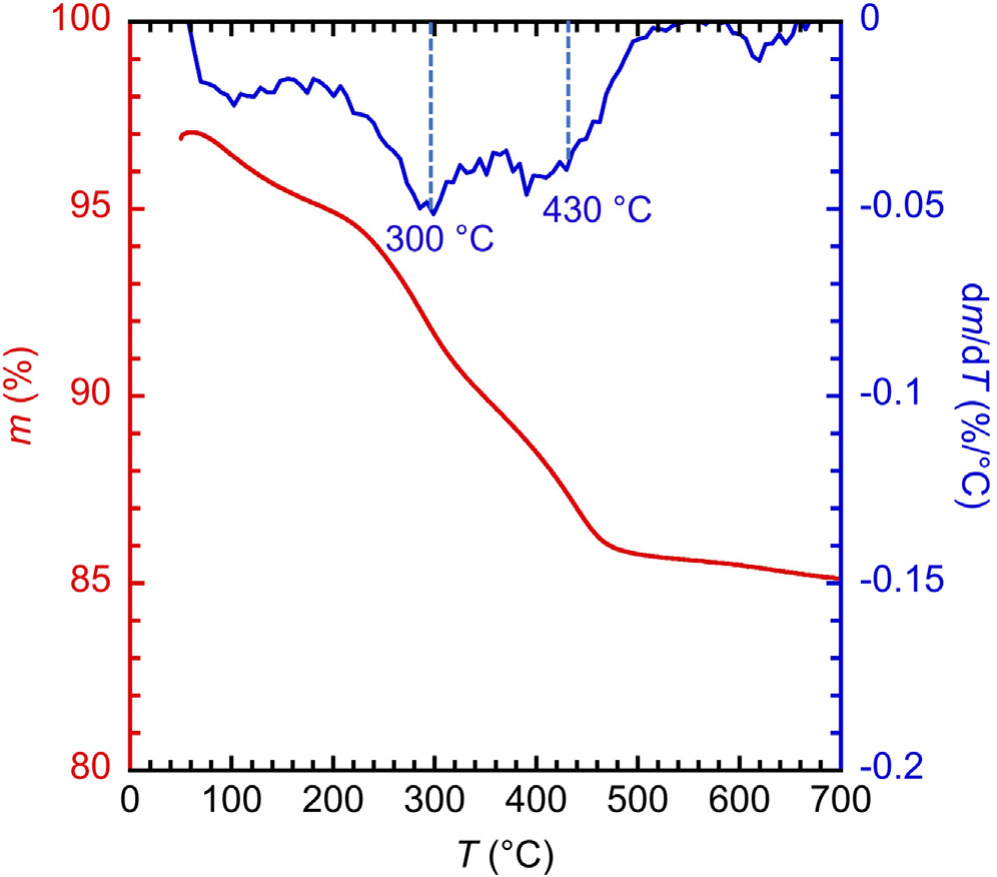
TGA (red) and corresponding DTGA (blue) for the solid residue recovered from PE depolymerization catalyzed by Pt/Cl-Al_2_O_3_

To characterize solid hydrocarbons, TGA is performed in air while ramping the temperature at a heating rate of 10°C min^−1^ from 50°C to 700°C. Intact PE is oxidized at ca. 300°C, while oxidation of coke requires higher temperatures.^20^ The peak positions are readily identified in the derivative data. TGA and DTGA of the insoluble solid recovered from the conversion of PE (0.120 g, *M*_w_ = 3.5 × 10^3^ g mol^–1^, *Ð* = 1.9) catalyzed by Pt/Cl-Al_2_O_3_ (0.200 g, 1.5 wt % Pt, 1.4 wt % Cl) after 8 h at 280°C under Ar shows the two distinct mass loss events (Figure 8).

## Quantification and statistical analysis

In this section, we describe methods to quantify the overall mass balance as well as specific types of hydrocarbon products in the gas, liquid, and solid phases. It includes the method for calculating the selectivities of various types of hydrocarbons (aromatics vs. alkanes, etc.) in the heavy liquid products. We also describe how to assess the depolymerization rate, by determining the rate of C-C bond scission.

### Hydrocarbon distribution

#### Quantification of gas products (C_1_-C_8_)

1. Calculate the individual and total amounts of gas products based on the GC-FID analysis.
  a. Assign the peaks for each carbon number (*i*) according to their retention times (assigned by comparison to individual *norm*-alkane standards from C_1_ to *norm*-C_8_ ).
    i. Assign all peaks that elute between *norm*-C_i-1_H_2i_ and *norm*-C_i_H_2i+2_ to have carbon number C_i_.
      ***Note:*** The *norm*-alkane has the highest boiling point of all alkane isomers.
  b. Calculate the amount of propene added using ideal gas law (Equation 3), where *n*_propene_ is the moles of propene added as internal standard, *P* is the propene partial pressure, *V* is the injected volume, *R* is the ideal gas constant and *T* is the absolute temperature.
    (Equation 3) $$n_{\text{propene}}=\left(PV\right)\;/\;\left(RT\right)$$
    ***Note:*** Propene was selected as our calibration standard because we typically did not detect it among our gas phase products. If propene is a reaction product, it can still be used for calibration, but its yield will need to be quantified using the method of standard additions.
  c. Calculate the number of moles (*n*_i_) and mass (*m*_i_) contributed by each species to the gas phase products, using its peak area (*A*_i_) relative to that of propene (*A*_propene_) using Equations 4 and 5. The volume expansion factor, *V*_2_/*V*_1_, is the total volume (*V*_2_) including the reactor, Schlenk line, and flask, relative to the volume of the Schlenk flask (*V*_1_).
    (Equation 4) $$n_{i}=\left(\left(A_{i}/i\right)n_{\text{propene}}/\left(A_{\text{propene}}/3\right)\right)\times \left(V_{2}/V_{1}\right)$$
    (Equation 5) $$m_{i}=n_{i}\left(14i+2\right)$$
    ***Note:*** It is reasonable to assume the same relative carbon response factor for all hydrocarbons in GC-FID analysis.^21^
  d. Calculate the total moles and the total mass of gas products by summing the contributions of each gas phase species.

#### Quantification of volatile liquids (C_7_-C_11_) and heavy (less volatile or non-volatile) liquids (C_>11_)

2. Calculate the individual and total amounts of volatile liquid product based on the GC-FID analysis.
  a. Assign carbon numbers to each peak according to its retention time, by comparison to the retention times for a standard mixture of *norm*-alkanes (C_7_-C_40_).
    i. Assign all peaks eluting between *norm*-C_i-1_C_2i_ and *norm*-C_i_C_2i+2_ to have carbon number *i.*
  b. For each carbon number in the region corresponding to C_7_-C_11_ (Figure 3), calculate the difference in peak areas (*A*) before and after solvent removal by evaporation. Calculate the moles of each species with a given carbon number species (*n*_i_) using the external standard method according to Equation 6, where *V*_0_ is the solution volume and *f* is the relative carbon response factor.
    (Equation 6) $$n_{i}=A_{i}\;V_{0}\;/\;\left(f\;i\right)$$
    ***Note:****norm*-octane in CH_2_Cl_2_ can be used as an external standard. A calibration curve is constructed using the peak areas for *norm*-octane at different concentrations.
    ***Note:*** It is reasonable to assume the same relative carbon response factor (*f*) for all hydrocarbons in GC-FID analysis.^21^
  c. Estimate the mass of each species with a given carbon number (*m*_i_), assuming its molecular weight to be that of the alkane (Equation 7).
    (Equation 7) $$m_{i}=n_{i}\left(14i+2\right)$$
  d. Calculate the mass yield of volatile liquids by summing the difference in masses for each carbon number in the C_7_-C_11_ region before and after solvent removal by evaporation.
3. Calculate the total amount of heavy liquid products.
  a. Obtain the mass of the heavy liquid by weighing the liquid remaining after solvent evaporation.
  b. Calculate the number of moles of heavy liquid using Equation 8, where *n*_heavy liq_ and *m*_heavy liq_ refer to the moles and mass of heavy liquid products, respectively.

(Equation 8) $$n_{\text{heavy}\;\text{liq}}=m_{\text{heavy}\;\text{liq}}/M_{n,\text{heavy}\;\text{liq}}$$

Here, *M*_n, heavy liq_ is the number-averaged molecular weight of the heavy liquid products, measured using GPC with PE calibration standards and RI detection.

4. Calculate the total liquid mass yield (wt %) using Equation 9, where *m*_volatile liq_ and *m*_initial PE_ refer to the mass of volatile liquids obtained in step 2d and the initial mass of PE, respectively.

(Equation 9) $$\text{Liquid}\;\text{yield}\;\left(\text{wt}\;\%\right)=\left(m_{\text{volatile}\;\text{liq}}+m_{\text{heavy}\;\text{liq}}\right)/m_{\text{initial}\;\text{PE}}\times 100\%$$

#### Quantification of the solid carbon residue

5. Obtain the yield of solid carbon residue by weighing the recovered solid and subtracting the catalyst mass, Equation 10, where *m*_solid residue_ and *m*_catalyst_ refer to the masses of solid carbon residue and catalyst, respectively.

(Equation 10) $$\text{Insoluble}\;\text{hydrocarbons}\;\left(\text{wt}\;\%\right)=\left(m_{\text{solid}\;\text{residue}}-m_{\text{catalyst}}\right)/m_{\text{initial}\;\text{PE}}\times 100\%$$

Here, we assume no mass loss of catalyst compared to the initial charge.

6. Calculate the individual masses of unreacted PE and coke in the recovered solid, using the TGA results (Figure 8).
  a. Calculate the contribution of unreacted PE (*m*_unreacted PE_%) to the recovered solid, using the percentage of mass loss between 200°C (*m*_200_%) and 350°C (*m*_350_%) in the TGA, as shown in Equation 11.
    (Equation 11) $$m_{\text{unreacted}\;\text{PE}}\%=m_{200}\%-m_{350}\%$$
  b. Calculate the contribution of coke (*m*_coke_%) to the recovered solid, using the percentage of mass loss between 350°C (*m*_350_%) and 600°C (*m*_600_%) in the TGA, as shown in Equation 12.
    (Equation 12) $$m_{\text{coke}}\%=m_{350}\%-m_{600}\%$$
  c. Calculate the contribution of the solid carbon residue relative to catalyst, using Equation 13.
    (Equation 13) $$m_{\text{carbon}\;\text{residue},\text{relative}\;\text{to}\;\text{catalyst}}\%=\left(m_{\text{unreacted}\;\text{PE}}\%+m_{\text{coke}}\%\right)/m_{600}\%\times 100\%$$
    ***Note:*** Since no mass loss was observed by TGA between 600°C and 700°C (Figure 8), coke was assumed to be fully oxidized below 600°C and the remaining mass (*m*_600_%) was considered to correspond to the catalyst. Depending on its structure, complete oxidation of coke may require temperatures higher than 600°C.
  d. Use the results from step 6c to obtain the percentage of mass of solid carbon residue (unreacted PE and coke) relative to initial PE, Equation 14.
    (Equation 14) $$m_{\text{carbon}\;\text{residue}}\%=m_{\text{carbon}\;\text{residue},\text{relative}\;\text{to}\;\text{catalyst}}\%\times \left(m_{\text{catalyst}}/m_{\text{initial}\;\text{PE}}\right)$$
  e. Check that the mass of carbon residue derived from the TGA measurement is consistent with the total solid hydrocarbon mass obtained directly by weighing in step 5 (Equation 10).

### Average rate of C-C bond scission

7. Calculate the number of C-C bond scission events using Equation 15, where *N*(0) is the number of moles of PE chains initially, and *N*(t) is the number of moles of hydrocarbons (polymer chains and small molecules) present at time *t.* Δ*N* therefore represents number of moles of new chains, which is equal to the moles of C-C bond scission events.
  (Equation 15) ΔN=N(t)–N(0)
  a. Calculate *N*(t) by summing the number of moles of hydrocarbons present in the gas and liquid phases (Table 1) and adding the contributions from the solid phase using Equation 16, where *n*_solid_ is the number of moles of chains present in the solid residue.
    (Equation 16) $$N\left(t\right)=\sum_{1}^{11}\;n_{i}+n_{\text{heavy}\;\text{liq}}+n_{\text{solid}}$$

**Table 1:** Sample hydrocarbon distribution from conversion of PE catalyzed by Pt/F-Al_2_O_3_

Carbon number (*i*)^a^	Gases (μmol)	Volatile liquids (μmol)	Heavy liquids (μmol)	Solid residue (μmol)	Total (μmol)
1	34.4	-	-	-	34.4
2	11.7	-	-	-	11.7
3	32.3	-	-	-	32.3
4	41.5	-	-	-	41.5
5	27.7	-	-	-	27.7
6	16.0	1.3	-	-	17.3
7	7.7	4.3	-	-	12.0
8	2.6	19.1	-	-	21.7
9	-	12.7	-	-	12.7
10	-	8.4	-	-	8.4
11	-	4.6	-	-	4.6
$\bar{i}$ = 24	-	-	231.3	-	231.3
$\bar{i}$ = 132	-	-	-	7.8	7.8

a Reaction conditions: PE (0.120 g, *M*_w_ = 3.5 × 10^3^ g mol^–1^, *Ð* = 1.9), Pt/F-Al_2_O_3_ (0.200 g, 1.6 wt % Pt, 0.8 wt % F), Ar, (280 ± 5)°C, 8 h.
    Here, *n*_solid_ is estimated as *m*_solid_/*M*_n,PE_, where *m*_solid_ refers to the mass of solid residue and *M*_n,PE_ is the initial number-averaged molecular weight of PE.
    ***Note:*** Using *M*_n,PE_ to calculate the number of chains in the solid residue is inaccurate if the residue contains partially depolymerized, but still insoluble, polymer chains. However, these chains contribute little to the number of chain cleavage events, relative to the gas and liquid products, therefore the error is small relative to other uncertainties inherent in the method.
    ***Note:*** If the mass balance is incomplete (i.e., less than 100 wt %), the missing mass must be assigned to one or more hydrocarbon components prior to using Equation 16. We assumed the missing mass to be unreacted PE. If a different assumption is made, the masses of the other presumed contributions to the total product mass should be scaled as appropriate.
    ***Note:*** Typically, the overall mass balance is 90 wt % or better using the mass recovery method described above. A poor mass balance will lead to a large error in the calculation of number of moles of C-C bond scission events.
  b. Calculate *N*(0) using Equation 17, where *m*_initial PE_ is the initial mass of PE and *M*_n, PE_ is its initial number-averaged molecular weight.
    (Equation 17) $$N\left(0\right)=m_{\text{initial}\;\text{PE}}/M_{n,\text{PE}}$$
8. Calculate the average rate of C-C bond scission (*r*_C-C_) using Equation 18.

(Equation 18) $$r_{C-C}=\Delta N/t$$

***Note:*** This approach assumes the rate law is pseudo-zeroth-order in the number of C-C bonds, which is appropriate for our reaction. If the reaction order is not zero, Equation 18 will still give the average rate. However, the numerical value will depend on the time interval chosen for the measurement.

### Assessment of liquid hydrocarbon product types and selectivity quantification

9. Identify the specific types of hydrocarbons present in the liquid products using ^1^H NMR.
  a. Identify aliphatic protons (H_ali_) via their signals in the range δ 0.5–2.0 ppm.
  b. Identify aromatic protons (H_arom_) by their signals in the range δ 6.5–9.0 ppm.
  c. Identify the aliphatic protons (H_α_) of the alkyl substituents of any aromatic ring (benzene, naphthalene, etc.) at the carbon directly bonded to the ring by their distinctive signals in the region δ 2.0–3.5 ppm.

***Note:***^1^H NMR analysis does not readily differentiate naphthenes from alkanes, or alkyltetralins from alkylbenzenes. For simplicity, the possible naphthenes and alkyltetralins contributions are simply combined with the contributions of alkanes and alkylbenzenes, respectively, in calculating alkane and aromatic selectivities.

***Note:*** Further identification of individual molecular components can discerned from the mass (FD-MS)^6^ and mass fragmentation (GC-EIMS) patterns for alkanes, cycloalkanes, alkylbenzenes, alkylnaphthalenes.

**Figure 9:**
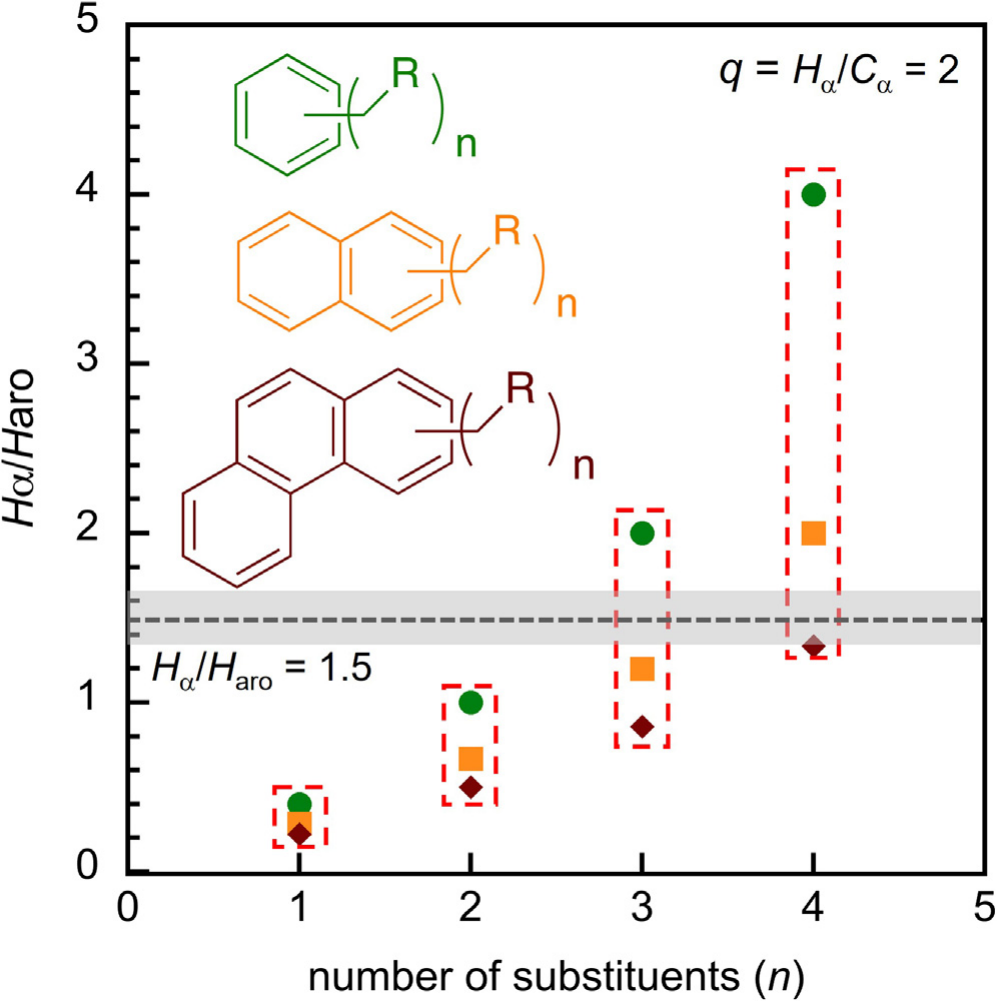
*H*_α_/*H*_arom_ ratios characteristic of alkylbenzenes (green), alkylnaphthalenes (orange), and alkylphenanthrenes (brown) with various numbers (*n*) of alkyl substituents (with average H_α_ content -C_α_H_2_R, corresponding to *q* = 2) The red boxes indicate the lower and upper bounds of *H*_α_/*H*_arom_ for a mixture of alkylaromatics. The black dashed line indicates the experimental ratio *H*_α_/*H*_arom_ = 1.5 obtained in our study, and the gray shaded area represents the error boundary from NMR integration (± 10%).

**Figure 10:**
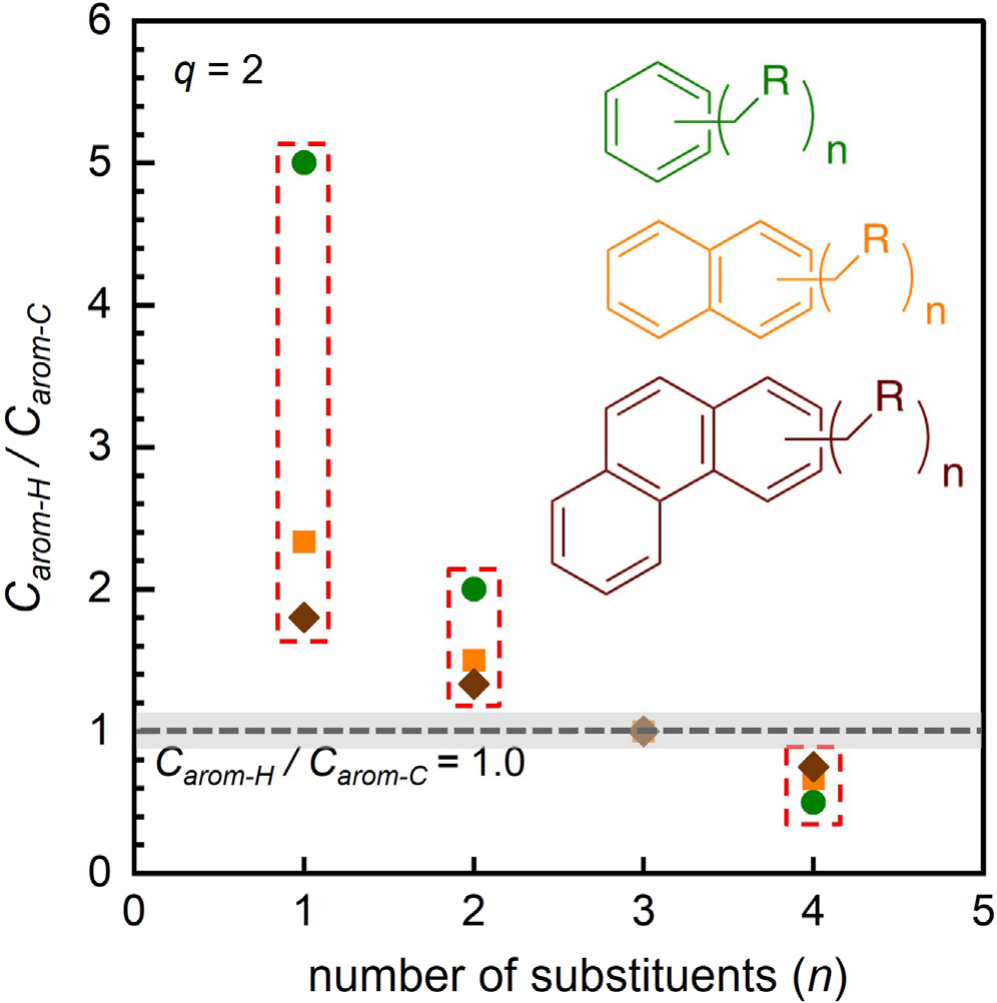
C_arom-H_/C_arom-C_ ratios characteristic of alkylbenzenes (green), alkylnaphthalenes (orange), and alkylphenanthrenes (brown) with various numbers (*n*) of alkyl substituents (with average H_α_ content -C_α_H_2_R, corresponding to *q* = 2) The red box indicates the lower and upper bounds of *H*_α_/*H*_arom_ for a mixture of alkylaromatics. The black dashed line indicates the experimental ratio C_arom-H_/C_arom-C_ = 1.0 obtained in our study, and the gray shaded area represents the error boundary from NMR integration (± 10%).
  d. If desired, predict the likely locations of the alkyl substituents based on the most plausible mechanism of aromatic ring formation.^1^
    ***Note:*** It is challenging to precisely identify the locations of substituents on aromatic rings present in a complex mixture of hydrocarbon products. Thermodynamics can provide guidance about the most stable substituent positions. For our methyldialkylaromatic products, we proposed the following plausible structures, based on the likely formation mechanism^1^: 1,2-dialkyl-3-methylbenzenes (**1**), 1,5-dialkyl-2-methylnaphthalenes (**2**), and 1,8-dialkyl-2-methylphenanthrenes (**3**), Scheme 1.

10. Estimate the aromatic yields and selectivities using ^1^H NMR.
  a. Identify each type of aromatic protons.
    i. Assign signals in the range δ 6.5–7.5 ppm (*H*_1_) to the aromatic protons of alkylbenzenes, alkylnaphthalenes, and polycyclic alkylaromatics.
    ii. Assign signals in the range δ 7.5–8.3 ppm (*H*_2_) to the aromatic protons of alkylnaphthalenes and polycyclic aromatics.
    iii. Assign signals in the range δ 8.3–9.0 ppm (*H*_3_) to the aromatic protons of polycyclic aromatics.
      ***Note:*** More information about the types of aromatic ring structures present in the liquids can be obtained by mass spectrometry and UV-vis spectroscopy. For example, three-ring structures were assessed as likely alkylphenanthrenes rather than alkylanthracenes and the presence of larger fused aromatics (e.g., chrysenes) was deemed unlikely on the basis of the UV-vis spectra.^1^
  b. Quantify the relative amounts of alkyl substituent types present on the aromatic rings.
    i. Integrate the signals for methyl substituents (*H*_α,CH3_), which appear in the range δ 2.0–2.5 ppm.
    ii. Integrate the signals for methylene-linked substituents (*H*_α,CH2R_), which appear in the range δ 2.5–3.1 ppm.
    iii. Integrate the signals for methine-linked substituents (*H*_α, CHR2_), which appear in the range δ 3.1–3.5 ppm.
    iv. Calculate the average number of H_α_ per alkyl substituent (C_α_), *q*, using Equation 19.
      (Equation 19) $$q=H_{\alpha }/C_{\alpha }=\left(H_{\alpha ,\text{CH}3}+H_{\alpha ,\text{CH}2R}+H_{\alpha ,\text{CHR}2}\right)/\left(H_{\alpha ,\text{CH}3}/3+H_{\alpha ,\text{CH}2R}/2+H_{\alpha ,\text{CHR}2}\right)$$
  c. Estimate the average number of alkyl substituents per aromatic molecule.
    i. Calculate the ratio of H_α_ signals (in the region δ 2.0–3.5 ppm) to H_arom_ signals (in the region δ 6.5–9.0 ppm), *H*_α_/*H*_arom_, by integrating the corresponding regions.
    ii. Calculate the ratio of C_arom-H_ signals (proton-bearing aromatic carbons, in the region δ 118–130 ppm) to C_arom-C_ signals (in the region δ 130–150 ppm), C_arom-H_ / C_arom-C_, by integrating the corresponding regions.^18^
    iii. Determine the average number of alkyl substituents (*n*) by comparing the experimental ratio *H*_α_/*H*_arom_ and C_arom-H_ / C_arom-C_ to the predicted value for the major type of aromatic product (or products, as appropriate), according to the value of *q* found in step 10b. The example of *q* = 2 (i.e., on average, C_α_H_2_R substituents) is shown in Figures 9 and 10. For simplicity, we assume that each type of aromatic product (e.g., alkylbenzenes, alkylnaphthalenes) in the liquid product mixture has the same number of alkyl substituents (see limitations below). Thus, the lower and upper bounds of *H*_α_/*H*_arom_ for a mixture of alkylaromatics are enclosed in the red, dashed box (Figure 9). For *H*_α_/*H*_arom_ = 1.5, the number of substituents could be 3 or 4 (dashed line in Figure 9), depending on the relative concentrations of alkylbenzenes, alkylnaphthalenes, and alkylphenanthrenes. Combining this information with the experimental ratio C_arom-H_ / C_arom-C_ (where C_arom-H_ / C_arom-__C_ = 1.0 in our study), we estimate that each aromatic molecule has, on average, 3 alkyl substituents.
      ***Note:*** This method works best when *n* ≤ 4, or the major types of aromatic products are ≤ 3 rings. Alkylaromatics with more than 4 substituents or higher polycyclic aromatics are not considered here.

**Scheme 1:**
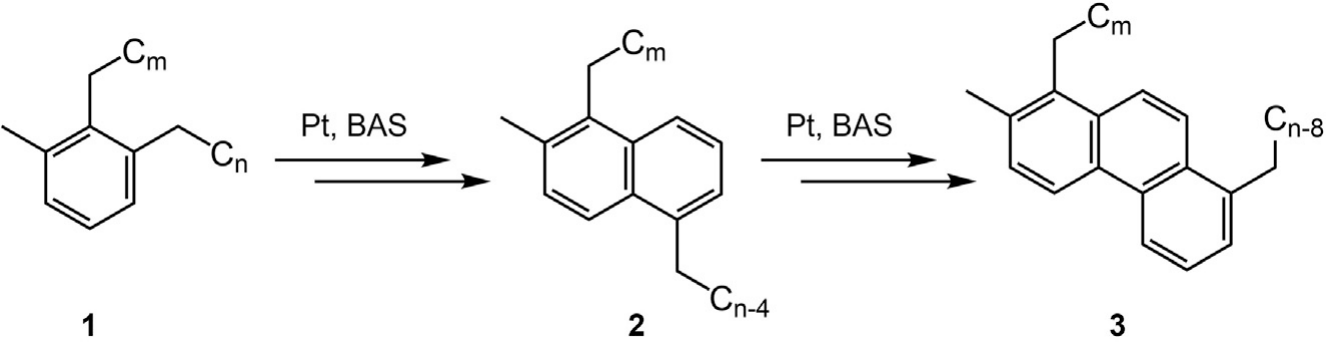
Plausible structures for methyldialkylaromatic products, proposed based on their likely formation mechanism
  e. Estimate the average carbon number for each type of hydrocarbon product, using MS
    i. Calculate the number-averaged carbon numbers for different types of hydrocarbons (e.g., alkanes, alkylbenzenes) using mass spectrometry with either hard ionization (GC-EIMS, with ion chromatograms isolated for individual classes of hydrocarbons)^22^^,^^23^^,^^24^^,^^25^ or soft ionization (FD-MS, with the corresponding molecular ion chromatograms). An example of data obtained using GC-EIMS is shown in Figure 6, where the individual ion chromatograms for alkylbenzene, alkylnaphthalene, alkylphenanthrene, and alkane products (Figures 6A–6D) were generated by summing the individual ion intensities (*I*) for each ion characteristic of a particular product group. The results allow us to calculate a separate number-averaged carbon number for each hydrocarbon type.
      ***Note:*** The characteristic ions for each species represented in Figure 6 are not fully exclusive. For example, alkylaromatics may also contribute ions to the alkane series (m/z = 43 + 57 + 71 + 85) due to the fragmentation of their alkyl chains. Additionally, alkyltetralins could contribute to the alkylbenzene ion series (m/z = 91 + 92 + 105 + 106 + 119 + 120 + 133 + 134). Consequently, this method is approximate.
      ***Note:*** Alkylbenzenes do not elute in precisely the same range as alkanes with the same carbon numbers. While branched alkanes typically elute just before the *norm*-alkane with the same mass, alkylbenzenes may elute either before or after the corresponding *norm*-alkane. Most alkylbenzenes have slightly longer retention times than the *norm*-alkanes, with retention times mostly between those of the *norm*-alkanes with carbon numbers *i* and *i*+1.^26^ Thus, when alkylbenzenes are a major component of the products, the average carbon number may be underestimated by ca. 1. This error is generally less than the accuracy of the analysis. Alkylnaphthalenes and alkylphenanthrenes generally elute later than alkybenzenes with the same carbon number, although they usually represent a small fraction of the alkylaromatic products and therefore do not have a large impact on the calculation of the average carbon number.
    ii. The calculations use one of two equations for each type of hydrocarbon, depending on the assumption made about the MS response factors (in the absence of individual response factors for each species). The example shown here applies to alkylbenzenes. Equation 20 assumes that the MS response factor is a function only of the type of hydrocarbon (e.g., alkane vs. alkylbenzene), not on its molecular weight (*M*). Alternatively, Equation 21 assumes that the MS response factor for each type of hydrocarbon also depends on its molecular weight. Typically, the estimated average carbon number calculated for each type of hydrocarbon is similar using either method (see Sun et al. for another sample calculation).^1^
      (Equation 20) $$\bar{i_{ben}}=\sum \left(i\cdot I_{ben,i}\right)/\sum I_{ben,i}$$
      (Equation 21) $$\bar{i_{ben}}=\sum \left(i\cdot \frac{I_{ben,i}}{M_{ben,i}}\right)/\sum \frac{I_{ben,i}}{M_{ben,i}}$$
      ***Note:*** Assign the carbon number of each species using *norm*-alkane standard compounds, according to the GC-FID method described above (see hydrocarbon distribution).
      As an alternative to step 10e.i, the average carbon number for heavier hydrocarbons can be identified by GPC using Equation 22, where $\bar{i}$ represents the number-averaged carbon number.
      (Equation 22) $$\bar{i}=M_{n,\text{heavy}\;\text{liquid}}/14$$
      ***Note:*** GPC analysis can be performed using both RI and UV detectors to reveal the distribution of aromatics (which absorb light) relative to the total hydrocarbon distribution (which includes alkanes that do not absorb UV light). The comparison may confirm that aromatics are evenly distributed across the molecular weight range of the liquid products, or reveal that aromatics are concentrated in a particular molecular weight range.
      ***Note:*** For simplicity, we assume the molecular formula to be C_n_H_2n_, based on the most intense signal at 1.2–1.7 ppm (CH_2_) in the ^1^H NMR spectrum.
  f. Estimate the contributions of each type of alkylaromatic to the ^1^H NMR signals.
    i. Integrate the relevant aromatic regions in the ^1^H NMR spectrum to obtain the ratios *H*_1_ : *H*_2_ : *H*_3_ (defined in step 10a above). These values are used in Equations (Equation 23), (Equation 24), (Equation 25), (Equation 26) to represent the molar amounts of each aromatic structure type. The relative molar selectivities (*S*_ben_, *S*_nap_, *S*_phe_) are obtained by solving Equations (Equation 23), (Equation 24), (Equation 25) simultaneously, as shown in Equation 26.
      (Equation 23) $$H_{1}=a\;n_{\text{ben}}+b\;n_{\text{nap}}+c\;n_{\text{phe}}$$
      (Equation 24) $$H_{2}=d\;n_{\text{nap}}+e\;n_{\text{phe}}$$
      (Equation 25) $$H_{3}=f\;n_{\text{phe}}$$
      (Equation 26) $$S_{\text{ben}}:S_{\text{nap}}:S_{\text{phe}}=\left(d\times f\;H_{1}-b\times f\;H_{2}+b\times e\;H_{3}-c\times d\;H_{3}\right)/\left(\text{adf}\right):\left(f\;H_{2}-e\;H_{3}\right)/\left(d\times f\right):H_{3}/f$$
      ***Note:*** The integrated intensities of the aromatic ^1^H NMR signals depend on the number and locations of the alkyl substituents on each aromatic ring (see Sun et al. for a sample calculation for methyldialkylaromatics).^1^
  g. Estimate the fraction of all aromatic protons, *H*_arom_/*H*_total_, by integrating the relevant regions of the ^1^H NMR spectrum, where *H*_total_ is the sum of *H*_ali_ and *H*_arom_. Equation 27 shows how the ratio is related to the chemical formulas and selectivities for each aromatic structure type.
    (Equation 27) $$\frac{H_{arom}}{H_{total}}=\frac{{3S}_{ben}+{5S}_{nap}+{7S}_{phe}}{{\left(2\bar{i_{ben}}-6\right)S}_{ben}+{\left(2\;\bar{i_{nap}}-12\right)S}_{nap}+{\left(2\bar{i_{phe}}-18\right)S}_{phe}+{\left(2\bar{i_{alk}}+2\right)S}_{alk}}$$
    ***Note:*** The numerical coefficients will change depending on the average number of alkyl substituents. Equation 27 is written for the specific case of trialkyl-substituted aromatics.
    ***Note:*** Calculating aromatic selectivity using the number-averaged carbon number is mathematically equivalent to a calculation using the full carbon number distribution instead. This equivalence is demonstrated below. The fraction of aromatic protons, *H*_arom_/*H*_total_, is obtained from the carbon number distribution in Equation 28:
    (Equation 28) $$\frac{H_{arom}}{H_{total}}=\frac{3\sum_{i}n_{ben,i}+5\sum_{i}n_{nap,i}+7\sum_{i}n_{phe,i}}{\sum_{i}\left[\left(2i-6\right)n_{ben,i}\right]+\sum_{i}\left[\left(2i-12\right)n_{nap,i}\right]+\sum_{i}\left[\left(2i-18\right)n_{phe,i}\right]+\sum_{i}\left[\left(2i+2\right)n_{alk,i}\right]}$$
    Since the number-averaged carbon number for each product type can be expressed in terms of the carbon number distribution (e.g., $\bar{i_{ben}}=\sum \left(i\cdot n_{ben,i}\right)/\sum n_{ben,i}$), each denominator term is rearranged as follows:
    (Equation 29) $$\sum_{i}\left[\left(2i-6\right)n_{ben,i}\right]=2\bar{i_{ben}}\sum_{i}n_{ben,i}-6\sum_{i}n_{ben,i}=\left(2\bar{i_{ben}}-6\right)\sum_{i}n_{ben,i}$$
    Thus, Equation 28 can be rewritten as Equation 30:
    (Equation 30) $$\frac{H_{arom}}{H_{total}}=\frac{3\sum_{i}n_{ben,i}+5\sum_{i}n_{nap,i}+7\sum_{i}n_{phe,i}}{\left(2\bar{i_{ben}}-6\right)\sum_{i}n_{ben,i}+\left(2\;\bar{i_{nap}}-12\right)\sum_{i}n_{nap,i}+\left(2\bar{i_{phe}}-18\right)\sum_{i}n_{phe,i}+\left(2\bar{i_{alk}}+2\right)\sum_{i}n_{alk,i}}$$
    The molar selectivity to alkylbenzenes (*S*_ben_) is defined in Equation 31 (molar selectivity for other components is defined similarly):
    (Equation 31) $$S_{ben}=\frac{\sum_{i}n_{ben,i}}{\sum_{i}n_{ben,i}+\sum_{i}n_{nap,i}+\sum_{i}n_{phe,i}+\sum_{i}n_{alk,i}}$$
    Finally, dividing each term in Equation 30 affords Equation 27.
  h. Assess the selectivity (*S* = *n*/*n*_total_) for alkane (*S*_alk_) and each type of alkylaromatics (*S*_ben_, *S*_nap_, and *S*_phe_) by solving Equations 26, 27, and 32 simultaneously.
    (Equation 32) $$S_{\text{ben}}+S_{\text{nap}}+S_{\text{phe}}+S_{\text{alk}}=1$$
    ***Note:*** The fractional selectivities sum to 1 (Equation 32) only if the liquid products consist only of various alkylaromatics and alkanes, as we have assumed.
  i. Calculate the molar yields of alkylbenzenes (*n*_ben_), alkylnaphthalenes (*n*_nap_), and alkylphenanthrenes (n_*phe*_) using Equation 33, and the total aromatics yield (*n*_arom_) using Equation 34.
    (Equation 33) $$n_{\text{ben}},n_{\text{nap}}\;\text{or}\;n_{\text{phe}}=\left(S_{\text{ben}},S_{\text{nap}}\;\text{or}\;S_{\text{phe}}\right)\;\left(m_{\text{heavy}\;\text{liq}}/M_{n,\text{liq}}\right)$$
    (Equation 34) $$n_{\text{arom}}=n_{\text{ben}}+n_{\text{nap}}+n_{\text{phe}}$$
11. As an alternative to the method described in step 10, estimate aromatic yields and selectivities using quantitative ^13^C NMR.
  a. Identify signals for aromatic and aliphatic carbons.
    i. Assign and integrate signals for aliphatic carbons (*C*_ali_) in the range δ 5–50 ppm.
    ii. Assign and integrate signals for aromatic carbons (*C*_arom_) in the range δ 118–150 ppm.
  b. Establish the potential contributions of alkylaromatic carbons to the ^13^C NMR signals using Equation 35.
    (Equation 35) $$C_{\text{arom}}=6\;n_{\text{ben},C}+10\;n_{\text{nap},C}+14\;n_{\text{phe},C}$$
    ***Note:*** The use of *C* in the subscripts of Equations (Equation 35), (Equation 36), (Equation 37) distinguishes the amounts and selectivities of different hydrocarbons obtained by ^13^C NMR analysis from those obtained by ^1^H NMR analysis, which are identified with a subscript *H* in Equation 37 below.
  c. Establish the relative contributions of the carbons present in each type of aromatic compound to the ^13^C NMR signals of all carbons using Equation 36, where *C*_total_ is the sum of *C*_ali_ and *C*_arom_.
    (Equation 36) $${C_{\text{total}}=n}_{ben,C}\cdot \bar{i_{ben}}+n_{nap,C}\cdot \bar{i_{nap}}+n_{phe,C}\cdot \bar{i_{phe}}+n_{alk,C}\cdot \bar{i_{alk}}$$
  d. Calculate the fraction of aromatic carbons, *C*_arom_/*C*_total_, based on the integration of the quantitative ^13^C NMR spectrum. The integrated ratio can be set equal to that established from the chemical formulas and molar yields for each aromatic structure type, in accordance with Equation 37.
    (Equation 37) $$\frac{C_{arom}}{C_{total}}=\frac{6\;n_{ben,C}+10\;n_{nap,C}+14\;n_{phe,C}}{n_{ben,C}\cdot \bar{i_{ben}}+n_{nap,C}\cdot \bar{i_{nap}}+n_{phe,C}\cdot \bar{i_{phe}}+n_{alk,C}\cdot \bar{i_{alk}}}$$
    ***Note:*** Since quantitative ^13^C NMR does not readily differentiate between types of aromatic rings, the molar ratios of the individual aromatic types must be assumed to be the same as those obtained from ^1^H NMR analysis.
  e. Calculate the selectivity for alkanes and for each aromatic structure type by solving Equations 32 and 37 simultaneously.
  f. Compare the total aromatic selectivity obtained using quantitative ^13^C NMR (or Equations 32 and 37) to that obtained from the ^1^H NMR analysis described above (Steps 10f-h and Equations 26, 27, and 32).

## Limitations

This protocol considers polyolefin depolymerization product mixtures that contain alkylaromatics (alkylbenzenes, alkylnaphthalenes and alkylphenanthrenes) and alkanes as the major components. Other types of hydrocarbons including naphthenes, alkyltetralins, and compounds with multiple, unfused aromatic rings are potential contributors to ^1^H NMR signals. For the purpose of this study, any naphthenes were combined with alkanes and all compounds containing aromatic rings were combined with alkylbenzenes in the calculations of selectivities towards alkanes and aromatics. However, if their contributions are significant, it could affect the corresponding calculations.

The locations of alkyl substituents on aromatic rings can be proposed based on plausible mechanisms for their formation (Scheme 1).^1^ The number of alkyl substituents is assumed to be similar for each aromatic structure type, based on the depolymerization mechanism. This assumption may not accurately represent the precise contributions of aromatic protons from each structure type. Characterization of the number of substituents and their positions for each aromatic structure type would improve the accuracy of the calculations. This characterization might be accomplished using GCxGC or other advanced separation methods, if available.

The number-averaged carbon number $\bar{i}$ for each type of hydrocarbon determined by mass spectrometry based on fragmentation patterns is semi-quantitative, because hydrocarbons with different structure types and chain lengths may have different ionization efficiencies in the mass spectrometer.

## Troubleshooting

### Problem 1

Unclosed mass balance due to incomplete recovery of gas products in Step 7 of the section titled “recovery and quantification of hydrocarbon reaction products” under “step-by-step method details.” This problem leads to an error in the calculation of number of C-C bond scission events.

### Potential solution

Check for leaks before expanding the gas from the reactor to the Schlenk flask. Very small leaks in the reactor, glass joints, rubber septa or the syringe connecting the reactor to the Schlenk collection flask via the Schlenk line can cause significant gas losses. Therefore, the reactor itself and each part of the set-up should be carefully leak-checked.

### Problem 2

Unclosed mass balance due to loss of volatile liquid products to the filtration in Step 13 of the section titled “recovery and quantification of hydrocarbon reaction products” under “step-by-step method details.” This problem also introduces error in the calculation of number of C-C bond scission events.

### Potential solution

Either direct distillation or cold filtration of the liquid products after reaction may be performed as an alternative to vacuum filtration to recover the volatile liquid products.

### Problem 3

Inability to quantify C_5_-C_7_ hydrocarbon products in step 2 of the section titled “hydrocarbon distribution” under “quantification and statistical analysis,” due to overlap of their signals in the GC-FID with the solvent signal. This issue reduces the apparent recovery and underestimates the number of C-C bond scission events.

### Potential solution

Methylene chloride can be substituted by *norm*-decane for extraction in an otherwise duplicate experiment. The mass of light hydrocarbons and the number of C-C bond scission events can then be evaluated by combining results for the different hydrocarbon ranges accessible using each extracting solvent.

### Problem 4

Incomplete mass balance as a result of a leak in the reactor for Step 1 of the section titled “polyethylene depolymerization” under “step-by-step method details.” A leaky reactor may result in a poor mass balance and potential exposure of the catalyst to air, which would affect reactivity.

### Potential solution

Since the depolymerization reaction is conducted under 1 atm of inert gas, it might be difficult to detect a leak in the reactor during reaction. Therefore, the reactor can be checked for leaks prior to use by pressurizing the reactor (> 1 atm) with inert gas and monitoring for pressure drop. The pressure should stabilize for an extended period. A leak typically happens at the connection between the upper part of the reactor and the reactor’s vessel. Replacing the O-rings should remedy a leaky reactor.

### Problem 5

Usage of methylene chloride (a hazardous chemical) as the extracting solvent in Steps 11–15, 17 and 19 of the section titled “recovery and quantification of hydrocarbon reaction products” under “step-by-step method details.”

### Potential solution

Hexane and ethyl acetate can be used as alternatives for extraction of the liquid product from the depolymerization reaction.

## Resource availability

### Lead contact

Further information and requests for resources and reagents should be directed to and will be fulfilled by the lead contact, Mahdi M. Abu-Omar (mabuomar@ucsb.edu).

### Materials availability

This study did not generate new unique materials.

## Data Availability

The published article (Sun et al.)^1^ and its extensive Supplemental Information include all data generated and analyzed during this study. No code is reported in this work.
